# Suppression, Maintenance, and Surprise: Neuronal Correlates of Predictive Processing Specialization for Musical Rhythm

**DOI:** 10.3389/fnins.2021.674050

**Published:** 2021-08-27

**Authors:** Ulvhild Færøvik, Karsten Specht, Kjetil Vikene

**Affiliations:** ^1^Department of Biological and Medical Psychology, University of Bergen, Bergen, Norway; ^2^Department of Education, The Arctic University of Norway, Tromsø, Norway; ^3^Mohn Medical Imaging and Visualization Centre, Haukeland University Hospital, Bergen, Norway

**Keywords:** musical rhythm and beat processing, temporal cortex, predicitve maintenance, supression, surprisal

## Abstract

Auditory repetition suppression and omission activation are opposite neural phenomena and manifestations of principles of predictive processing. Repetition suppression describes the temporal decrease in neural activity when a stimulus is constant or repeated in an expected temporal fashion; omission activity is the transient increase in neural activity when a stimulus is temporarily and unexpectedly absent. The temporal, repetitive nature of musical rhythms is ideal for investigating these phenomena. During an fMRI session, 10 healthy participants underwent scanning while listening to musical rhythms with two levels of metric complexity, and with beat omissions with different positional complexity. Participants first listened to 16-s-long presentations of continuous rhythms, before listening to a longer continuous presentation with beat omissions quasi-randomly introduced. We found deactivation in bilateral superior temporal gyri during the repeated presentation of the normal, unaltered rhythmic stimulus, with more suppression of activity in the left hemisphere. Omission activation of bilateral middle temporal gyri was right lateralized. Persistent activity was found in areas including the supplementary motor area, caudate nucleus, anterior insula, frontal areas, and middle and posterior cingulate cortex, not overlapping with either listening, suppression, or omission activation. This suggests that the areas are perhaps specialized for working memory maintenance. We found no effect of metric complexity for either the normal presentation or omissions, but we found evidence for a small effect of omission *position*—at an uncorrected threshold—where omissions in the more metrical salient position, i.e., the first position in the bar, showed higher activation in anterior cingulate/medial superior frontal gyrus, compared to omissions in the less salient position, in line with the role of the anterior cingulate cortex for saliency detection. The results are consistent with findings in our previous studies on Parkinson’s disease, but are put into a bigger theoretical frameset.

## Introduction

New musical forms and experiments often challenge established structural forms in composition or reshape old ones into new uses, reordering fundamental musical building blocks to challenge the perception of music. The challenge of studying such diverse music in a principled and systematic manner within a context of neuroscience demands first to establish some fundamental and perhaps common mechanisms in music perception, a work that has been flourishing in the last decades. In this article, we focus on rhythm, one of these fundamental building blocks. Within new music, some artists work with rhythmic entropy while others try to dispel rhythm altogether. Insights into some basic mechanisms of the “listening apparatus” in our nervous system as they pertain to perception of musical rhythms might perhaps still be of use to both researchers and artists, either as a starting point for more advanced research or as a starting point of artistic defiance.

In short, auditory repetition suppression and omission activation, which this study address, are opposite neural phenomena and manifestations of principles of predictive processing. Repetition suppression is the reduction of neuronal activity during listening to a repeated sound that is *present*, omission activation is the increased neuronal activity that occurs when an expected sound is *not* present. Listening activates bilateral superior temporal cortices, independent of whether we are exposed to tones, words, animal, and instrumental sounds (Specht and Reul, 2003), while prolonged listening to an unchanging sequence of sounds quickly leads to a deactivation of neural activity, a phenomenon known as repetition suppression (Grill-Spector et al., 2006). Auditory repetition suppression is a robust, experience-dependent adjustment of neural functions (Grotheer and Kovacs, 2016), predictability (Costa-Faidella et al., 2011; Cacciaglia et al., 2019), and prior expectation (Summerfield et al., 2008; Todorovic et al., 2011). It is modulated by a range of factors, such as time scales of presentation rates, sequence position and stimuli similarities (Kovács and Schweinberger, 2016), and stimuli-specific characteristics (Linke et al., 2011), and is also task-dependent (Arnott et al., 2005). Repetition suppression also neatly demonstrates principles of predictive coding (Friston, 2005; Baldeweg, 2006; Auksztulewicz and Friston, 2016), where repetition suppression biases the activity in sensory cortices (Sreenivasan et al., 2014). For auditory stimuli, repetition suppression can be seen in the temporal cortices, including Herschel’s gyrus (HG), superior temporal gyrus (STG), and middle temporal gyrus (MTG) (Auksztulewicz and Friston, 2016; Cacciaglia et al., 2019).

Repetition suppression is related to working memory mechanisms, where attenuation of neural activity can be interpreted as a minimization of activity needed in a working memory maintenance stage (Kumar et al., 2016) through the suppression of irrelevant information (Linke et al., 2011; Ahveninen et al., 2017). It is, however, unclear whether working memory is dependent on persistent neural activity (for maintaining the information) (Huang et al., 2016) or on transient reorganization of synaptic weights (D’Esposito and Postle, 2015) in representational states (Myers et al., 2017; Sreenivasan and D’Esposito, 2019) or a combination of the two (Sreenivasan et al., 2014). A potential theoretical (or actual) difference between these two descriptions—working memory as either persistent or transient neural states [or what within a predictive coding framework can be called “representation units” (Clark, 2013)]—could lie in a distinction between auditory sensory memory (shorter low-level sensory cortical retention intervals) and higher-level working memory network organization (Nees, 2016). Different cortical activation for simple sequence processing and more complex, task-specific working-memory maintenance could point to such nuances (Brechmann et al., 2007). Neural correlates for auditory working memory have been shown in temporal cortices, including STG, HG, and planum temporale (PT) (Brechmann et al., 2007; Kumar et al., 2016). Furthermore, distinct neural differences between perceptual processing and active working memory tasks for melody and pitch (Zatorre et al., 1994), separate neural correlates for duration-based and beat-based auditory timing (Teki et al., 2011), and differentiations for melody and rhythm have been shown—with the right inferior frontal gyrus and insula particularly involved (Jerde et al., 2011).

Within a predictive coding framework, auditory prediction errors (Friston, 2005) must depend on working memory or sensory memory mechanisms, since they occur when an unchanging and predictable sequence of sounds suddenly changes, i.e., when predictions and expectations are breached, or when the incoming sensory signal does not match the “representation unit” (Clark, 2013); what we will henceforth call the *representational maintenance*. The concept of prediction errors [or “surprisal” (Clark, 2013)] draws on findings in EEG/ERP-studies on mismatch negativity (MMN) (Naatanen et al., 1978; Kompus et al., 2015). MMN potentials are measurable neural spikes triggered by deviant and rare stimuli in a chain of standard stimuli, where the difference between the deviant and the standard stimuli are proportional to the deviance [see (Näätänen, 1992) for a review]. This difference is the *mismatch* or prediction error (Friston, 2005). In fMRI, as in EEG/ERP studies, the size of activation reflects the magnitude of the MMN deviant (Mathiak et al., 2002; Liebenthal et al., 2003; Eichele et al., 2008). Omissions are a particularly interesting type of deviant stimuli, where omission activation (Raij et al., 1997; Wacongne et al., 2011) describes cortical activation as a result of *missing* stimuli in a predictable sequence of sounds, and can therefore be assumed to be generating internal responses based solely on expectancy or prediction, and not by a change in the deviant characteristics in the stimuli itself (Jongsma et al., 2005). As with repetition suppression, MMNs or prediction error magnitude is modulated by numerous factors (Näätänen, 1992), and as with repetition suppression and auditory working memory, specific parts of the temporal cortices have repeatedly been shown to be involved in the reporting of such prediction errors (Friston, 2005). Imaging and electrophysiological studies have consistently shown that a main source of MMN potentials is located in the intersection of STG, PT, and HG (Recasens et al., 2014), predominantly right lateralized (Tervaniemi et al., 2000; Opitz et al., 2002; Doeller et al., 2003; Rinne et al., 2005), also for omissions (Mustovic et al., 2003; Voisin et al., 2006; SanMiguel et al., 2013a, b), although one study has found omission activation predominantly on the left (Nazimek et al., 2013). Pertaining to our study, these cortical areas are sensitive to beat and pattern deviations, as shown in both EEG/ERP and fMRI studies (Tervaniemi et al., 2000; Opitz et al., 2002; Doeller et al., 2003; Mustovic et al., 2003; Rinne et al., 2005; Voisin et al., 2006; Nazimek et al., 2013; Recasens et al., 2014). Beat omission and positional saliency have been used to investigate rhythm and pattern-related phenomena with several imaging and neurophysiological techniques, indicating different levels of magnitude for salient and less salient metric beat positions, although findings are somewhat ambiguous (Winkler and Schroger, 1995; Jongsma et al., 2003, 2005, 2006; Ladinig et al., 2009; Wacongne et al., 2011; Salisbury, 2012; Bouwer et al., 2014; Damsma and van Rijn, 2016).

In the current study, we wanted to examine repetition suppression, representational maintenance, and omission activation during the perceptual processing of musical rhythms. In short, these three phenomena can be seen as manifestations of key principles in predictive processing frameworks, and musical rhythms are ideal stimuli to operationalize and demonstrate these principles because of their predictive, temporal nature (Vuust and Witek, 2014; Koelsch et al., 2019). Musical rhythms also facilitate operationalization of modulating factors such as contextual characteristics (simple or complex rhythms) and saliency (position of omission).

In addition, the neuronal mechanisms involved in the perception of musical rhythms are partly known, which makes it possible to compare our results with existing literature. Listening to rhythms activates cortical motor areas, such as premotor cortex and supplementary motor area (SMA), the basal ganglia, as well as large-scale networks across the brain (Grahn and Brett, 2007; Chen et al., 2008a, b; Bengtsson et al., 2009; Geiser et al., 2009, 2012; Grahn, 2009; Trost et al., 2014; Large et al., 2015).

We also wanted to examine the effect of pattern *complexity* on repetition suppression and omission activation and the effect of *positional saliency* on omission activation. To this end, two musical rhythms, one simple and one complex, were presented several times to the participants during scans. The first part of the stimuli presentation consisted of a short presentation (16 s) of continuous, unperturbed rhythmic repetition to examine repetition suppression, segueing into a longer continuous presentation of the same rhythm. In this second part, an overt target-detection task (with a quasi-randomly distributed deviant tone) was introduced to keep the participant attending to the rhythm, while quasi-randomly distributed beat omissions were used to covertly examine omission activation (see section “Materials and Methods” for more details on the stimuli and the paradigm).

Based on previous literature, we expected to see listening, repetition suppression, and omission activation in largely overlapping areas in the temporal cortices, with omission activation occurring in more posterior areas than suppression and maintenance. We hypothesized that representation maintenance would also occur in the temporal cortices, with additional activation of inferior frontal areas, insula, and premotor cortices. We hypothesized that complexity would differentially affect suppression, as the encoding stage presumably would be affected by a higher cognitive load for the complex rhythm. We also hypothesized that omission activation would be modulated by rhythmic context where the cognitive demand (i.e., higher neural activity in a representational maintenance) of the more complex rhythm would result in smaller omission sizes, and furthermore that *positional* saliency would modulate omission activation, where a more salient position (beat position number one) would show higher activation than the less salient position (beat position number two). Finally, we hypothesized that there would be an interaction between pattern complexity and beat position for omission activation.

## Materials and Methods

### Participants

Participants were recruited among Norwegian-speaking students enrolled at the University of Bergen (UiB). Fourteen participants underwent scanning with fMRI, but four were excluded due to head movement in the scanner. Analyses were done on the remaining 10 (6 females, mean age = 24.4). Eight were right-handed by self-report. The number of years participants had played instruments (outside mandatory music lessons in public school) was done by self-report. A participant was labeled as a musician if s/he had 5 or more years of consistent instrument practice, and 5 out of the 10 participants reached this target. Personal data were coded and stored offline and anonymity was assured. All procedures were approved by the Regional Committee for Medical and Health Research Ethics (REK no 2014/1915) and carried out in accordance with the code of Ethics of the World Medical Association, Declaration of Helsinki. Upon enrollment in the study, all participants gave written informed consent to participate in the study and were rewarded 50NOK for their participation.

### Stimuli

We used two musical rhythmical stimuli of different rhythmic complexity described elsewhere (Vikene et al., 2018, 2019). The stimuli consisted of deep, multilayered synthesizer bass sounds in two octaves and a sampled bass drum sound, to place the general character of the stimuli in a different frequency range than the Eigenfrequency of the scanner during the echo planar imaging (EPI) sequence. The first 8 bars (16 s) of each stimulus contained an alternating piano chord, at the first position to clearly mark the beginning of the bar. For each rhythm (simple/complex), these chords were composed in one major and one minor mode for listening variation (no tests where planned for the effect of mode). The remaining 44 bars/88 s of each stimuli were constructed with quasi-distributed overt deviant probe tones (consisting of a six-note up-shift of tonality, to keep the participants attending to the musical rhythms), and covert beat omissions. Probe tones were always placed on the first position of the bar, while omissions were placed in equal numbers on first and second positions. Each of the four versions of the stimuli blocks (simple/complex vs. major/minor) had six omissions. The omissions were either at the first or second position of the rhythmic patterns. In two versions of the blocks, three omissions were at the first position and three omissions were at the second position. In one version of the blocks, four omissions were at the first position and two were at the second position. In one version of the blocks, two omissions were at the first position and four were at the second position. The smallest time between two consecutive omissions was 8.5 s; the longest was 17.5 s. Because we were interested in the covert, or passive, detection of omission, and not active detection success, and because target detection of the probe tones involved button-pushing on a hand grip leading to motor area activity, no analysis was planned for the overt probe tone detection. All participants did, however, correctly detect all target tones. Stimuli were created using Steinberg Cubase 7 and presented in the scanner with EPrime 2.0 (Ver 2.3 Professional), which was also used to collect responses to the overt task.

### Experimental Design

**FIGURE 1:**
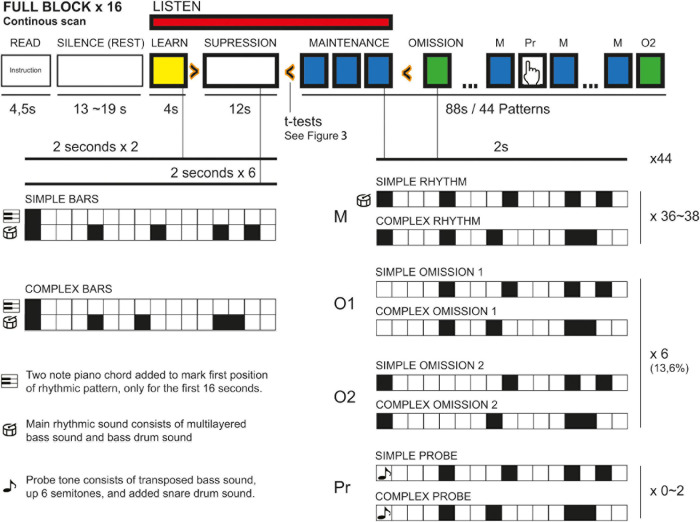
Construct of fMRI paradigm.

Participants were given earplugs and were placed comfortably in the scanner. They were given fMRI-compatible headphones with additional physical noise cancelation foamed ear plugs. Participants were also fitted with fMRI-compatible video goggles and a handgrip with buttons to respond to the overt target-detection attentional task. After initial structural scans and a 5-min-long resting-state fMRI scan (not part of this report), goggles were turned on and participants were given instructions for the study. Participants were told to keep eyes open and look at a cross in the middle of the screen and asked to press a button on the hand grip when the probe tone was detected. Instructions were followed by a short test run before scanning started. Before each trial, the same written instructions were repeated in the goggles (4.5 s), followed by a blank screen and silences ranging from 13 to 19 s. When the music stimuli began playing, a cross was presented in the goggles as a focus point to minimize head movement. Each sound file was presented twice during the scan, in randomized order between subjects. Total scan time for the paradigm was 33 min. See Figure 1 for an overview of the paradigm.

### Data Acquisition and Pre-processing

fMRI images were acquired using a 3-T scanner (GE Signa Excite 750) with a 32-channel coil. Repetition time (TR) for the EPI sequence was 1.5 s, echo time (TE) was 30 ms, voxel size was 3.44 × 3.44 × 5 with 28 slices interleaved, for 1325 volumes. Pre-processing steps included realignment (0.9 quality, 5 mm smoothing kernel, registered to first image with second-degree B-spline), unwarping (using 12 × 12), resliced to mean image, normalized to ICBM template (with 2mm^3^ voxel size), and smoothing with Gaussian kernel (5 mm^3^).

### First-Level Analysis

We aligned the onset of stimuli epochs (below) to 13th of the 28 interlaced slices. A high-pass filtering threshold was set at 1/249 Hz cutoff (calculated as the mean between onsets of the stimuli blocks). Single-subject data were analyzed by specifying a general linear model, and for the whole scan, movement-related variance (realignment parameters) was included in the model as six covariates of no interest.

Each block was modeled as follows: 4.5 s of on-screen instructions were labeled as “READ.” Silence periods (randomly assigned between 13 and 19 s) between each block were not segmented and thus served as contrast for all other epochs (“REST”). The whole block was segmented into 2-s bins, i.e., the total length of one whole rhythmic pattern. The first 4 s of each block were labeled “LEARNING”; the next 12 s, “SUPPRESSION.” In the following 88 s of each block, bins containing a probe tone were labeled “PROBE,” bins containing the normal, unperturbed version of the rhythmic pattern were labeled “MAINTENANCE,” while 2-s bins containing an omission were labeled according to the position of the omission (i.e., “OMISSION1” for the first position-omission). The 2-s bin immediately following an omission was labeled “NOT OF INTEREST” to avoid any secondary effects of the omission spilling into the segments labeled “MAINTENANCE.”

All blocks were divided into simple and complex rhythm, and segmentation of different epochs were labeled “SIMPLE” or “COMPLEX.” For example, the “LEARNING” epochs in the simple rhythm were called “SIMPLE_LEARNING”; for the omission in position 2 in the complex rhythm: “COMPLEX_OMISSION2.” First-level analysis produced 10 contrasts for “SIMPLE/COMPLEX” blocks, with “LEARNING/SUPPRESSION/MAINTENANCE/OMISSION (1/2)” epochs, all with REST epochs subtracted for the contrasts.

### Second-Level Analysis (Initial)

A full factorial analysis—akin to a repeated measures ANOVA—was conducted, with MUSICIAN (musician/non-musician), RHYTHM (“SIMPLE/COMPLEX”), and TYPE [“LEARNING/SUPPRESSION/MAINTENANCE/OMISSION (1/2)] as dependent factors. The analysis was examined with a threshold of family-wise error (FWE) correction for multiple comparisons at *p* < 0.05, with a minimal voxel-cluster size of at least 10 voxels. No main effect or interaction effects were found for neither MUSICIAN nor RHYTHM, but as expected, a main effect for TYPE was found. Since we had a clear hypothesis on omission complexity and position, we did, however, probe these comparisons through *t*-tests, but did not find any significant differences on omission, neither between rhythms nor positions at a FWE-corrected level. For completeness, we would nonetheless mention that at an uncorrected level (*p* < 0.001, cluster size of 100 voxels), we found higher activation in an area in the anterior cingulate cortex (ACC)/medial prefrontal gyrus (see Supplementary Figure 1 and Supplementary Table 1).

### Second-Level Analysis (Reduced Model)

Based on the lack of main and interaction effects for RHYTHM, and lack of significant *t*-test results on OMISSION, we decided to re-segment the data, dispelling of the division into two rhythms, as well as omission position. Furthermore, based on the lack of main and interaction effects of the categorical MUSICIAN group division, we instead included the number of years playing an instrument as a covariate in the analysis. A new full factorial analysis was therefore conducted using only TYPE as dependent factors, i.e., “LEARNING/LISTENING/MAINTENANCE/OMISSION.” A main effect for TYPE was found [*F*(1,31) = 18.36, *p* < 0.001], and we proceed to do *t*-tests for our planned comparisons.

## Results

**FIGURE 2:**
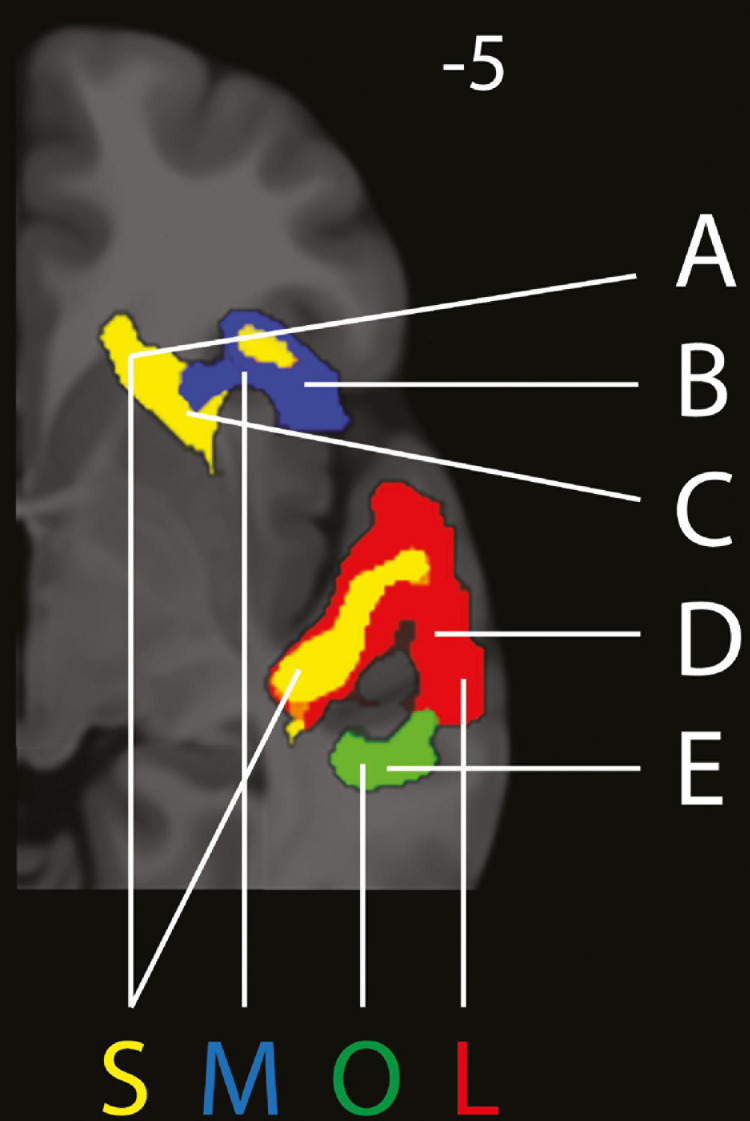
Excerpt from right hemisphere of separation of (in red) LISTENING (L), (in yellow) SUPRESSION (S), (in blue) MAINTENANCE (M) and (in green) OMISSION (O). A = Caudate Nucleus, B = Anterior Insula, C = Putamen, D = Superior Temporal Gyrus, E = Middle Temporal Gyrus. Panel at MNI z = –5 on the axial plane. (Figure made partly with MRIcroGL, with small cluster removal).

**FIGURE 3:**
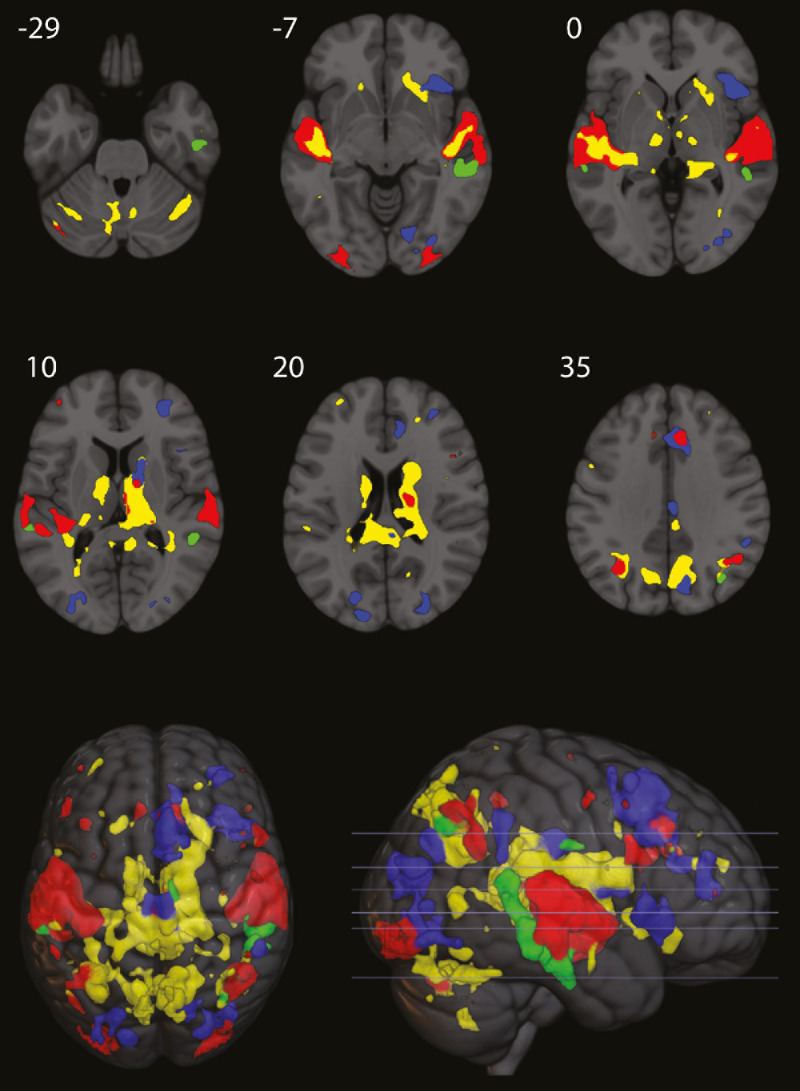
Red areas, overall activity during listening for the first 16 s. Yellow areas, where activity decreased in the last 12 s, compared to the first 4 s of listening. Blue areas, persistent activity for the remaining 88 s of listening (only for epochs of normal presentation of the rhythm). Green areas, beat omission activity in the middle temporal gyrus. Numbers refer to MNI coordinate on the axial plane (figure made partly with MRIcroGL, with small cluster removal).

All results are reported with a FWE-corrected threshold of *p* < 0.05 and at least 10 voxels per cluster. Figure 2 shows a detailed excerpt of the frontal right hemisphere of the findings listed below. Figure 3 shows a more detailed overview of the findings across the while brain.

### Overall Listening

For the combined LEARNING, SUPPRESSION, and MAINTENANCE bins, we found activations in large parts of bilateral superior and middle temporal gyri (STG/MTG), as well as bilateral angular gyri, SMA, cerebellum, and posterior areas [fusiform, occipital, posterior cingulate cortex (PCC)] (Table 1).

**TABLE 1:** General effect of listening (LEARNING + LISTENING + MAINTENANCE).

			**MNI**			
	**Region**	***X***	***Y***	***Z***	**Size**	***t***
R	Superior temporal gyrus	42	−20	−6	1146	11.97
	Extending to	50	−10	−10		10.75
	Middle temporal gyrus	56	−4	−8		10.32
L	Superior temporal gyrus	−46	−8	−6	1518	11.73
	Extending to	−44	−16	−8		11.34
	Middle temporal gyrus	−52	−16	−4		10.71
R	Angular gyrus	34	−84	−16	61	8.70
		32	−94	−16		6.20
		28	−94	−8		6.12
L	Cerebellum crus 1	−46	−72	−34	22	8.28
R	Angular gyrus	42	−56	28	50	7.86
L	Fusiform	−24	−96	−20	81	7.48
L	Inferior occipital cortex	−28	−88	−16		7.47
		−42	−88	−10		6.78
R	Supplementary motor area	0	18	48	29	7.32
		2	24	42		6.29
R	Posterior cingulate cortex	4	−22	28	49	7.22
L	Angular gyrus	−34	−64	36	49	7.19
		−36	−56	40		6.62
R	Supplementary motor area	10	24	36	24	6.81
R	Thalamus	4	−14	16	10	6.76
L	Middle temporal gyrus	−48	32	26	12	6.75
R	Inferior frontal gyrus	56	14	26	20	6.59
R	Inferior occipital cortex	28	−84	−4	10	6.52

*All results reported as *t*-tests with family-wise error (FWE) correction at *p* < 0.05, cluster size of 10 voxels. Coordinates listed without cluster size belong to the previous listed cluster. Size = number of voxels activated in the cluster.*

### Learning/Suppression

We contrasted the first 4 s of the introduction of music, LEARNING, by subtracting the following 12 s of SUPPRESSION. This showed more bilateral activations across many areas, including STG, angular gyri, and cerebellum (crus 1 and vermis 6), the basal ganglia (caudate nucleus and putamen), and thalamus, in the first 4 s (Table 2).

**TABLE 2:** Effect of Time (LEARNING > SUPPRESSION).

			**MNI**			
	**Region**	***X***	***Y***	***Z***	**Size**	***t***
R	Thalamus	6	−22	12	1650	10.38
	Caudate nucleus	12	−2	14		10.36
	Hippocampus	12	−28	8		9.7
R	Precuneus	8	−66	34	357	8.86
		10	−72	48		7.62
		12	−70	26		7.55
L	Precuneus	−10	−68	40	94	8.56
		−8	−76	44		6.26
R	Superior temporal gyrus	44	−20	−6	78	8.46
	Middel temporal gyrus	50	−12	−10		7.26
L	Angular gyrus	−32	−68	44	180	8.42
	Inferior parietal lobule	−32	−62	36		8.24
L	Thalamus	−12	−6	12	114	7.98
	Caudate nucleus	−14	0	24		6.11
L	Cerebellum crus 1	−4	−80	−20	26	7.8
L	Cerebellum crus 1	−44	−72	−34	16	7.72
L	Cerebellum 6	−30	−66	−26	38	7.62
L	Superior temporal gyrus	−44	−22	−4	182	7.62
	Inferior temporal gyrus	−40	−26	−18		7.6
	Middle temporal gyrus	−52	−24	0		7.22
R	Putamen	22	22	−4	57	7.61
R	Cerebellum crus 1	48	−48	−28	52	7.59
		38	−60	−28		7.46
L	Hippocampus	−28	−28	0	101	7.32
		−34	−32	8		6.76
	Thalamus	−22	−28	14		6.19
L	Superior temporal gyrus	−66	−22	10	39	7.26
		−60	−26	6		7.07
L	Planum temporale	−34	−44	8	25	7.26
L	Thalamus	−10	−14	2	24	7.12
L	Precuneus	−8	−78	54	17	6.69
R	Rectus	10	30	−12	15	6.56
R	Angular gyrus	40	−62	38	10	6.17

*All results reported as *t*-tests with family-wise error (FWE) correction at *p* < 0.05, cluster size of 10 voxels. Coordinates listed without cluster size belong to the previous listed cluster. Size = number of voxels activated in the cluster.*

### Maintenance

We subtracted the last of the 12 s of the initial listening (SUPPRESSION) from MAINTENANCE to examine which brain areas, after the initial listening period, showed more activation during maintenance of the rhythm throughout the remaining 88 s. The rationale behind this contrast was that after the first 4 s (LEARNING), the next 12 s (SUPPRESSION) consolidates the (reduced) neural activation related to the establishment of a predictive model of the rhythmic pattern, while MAINTENANCE represents additional areas needed to keep the rhythmic pattern in working memory pertaining to the overt task. Areas including SMA; caudate nucleus; anterior insula (AIN); superior, middle, and inferior frontal gyrus (SFG/MFG/IFG); and middle and posterior cingulate cortex, as well as parts of the occipital cortex, showed more activation during MAINTENANCE than during SUPPRESSION (Table 3).

**TABLE 3:** Maintenance of rhythmic patterns (MAINTENANCE > SUPPRESSION).

			**MNI**			
	**Region**	***X***	***Y***	***Z***	**Size**	***t***
R	Supplementary motor area	4	12	56	366	8.97
		2	20	54		8.88
	Middle cingulum cortex	10	24	36		8.03
R	Precuneus	12	−74	32	61	8.23
R	Anterior insula	34	28	−4	97	8.23
		42	16	−2		7.02
R	Posterior cingulum cortex	4	−28	26	147	8.16
		−4	−20	28		7.17
		12	−36	24		6.49
R	Caudate nucleus	14	−2	14	53	7.91
		16	8	12		6.54
R	Inferior frontal gyrus	50	20	4	21	7.39
R	Lingual	16	−78	−8	17	7.28
L	Superior occipital	−14	−92	20	26	7.21
R	Superior frontal	30	48	10	22	7.02
L	Middle occipital	−20	−78	20	30	6.91
R	Inferior occipital	34	−80	−14	31	6.77
		30	−82	−6		6.55
R	Middle frontal gyrus	46	18	42	10	6.77
R	Middle occipital	28	−84	20	17	6.49

*All results reported as *t*-tests with family-wise error (FWE) correction at *p* < 0.05, cluster size of 10 voxels. Size = number of voxels activated in the cluster.*

### Omission

We contrasted OMISSION by subtracting MAINTENANCE to examine which brain areas were activated during beat omissions. The rationale behind this contrast was that MAINTENANCE, as stated above, represents areas needed to keep the rhythmic pattern in working memory, while OMISSION represents the neural activation related to the prediction error triggered by the missing beat.

For OMISSION, more activation was seen, predominantly in the right MTG, extending from the inferior to the superior temporal gyrus. In addition, a smaller activation was seen in the left MTG as well as in the right angular gyrus (Table 4).

**TABLE 4:** Omission activation (OMISSION > MAINTENANCE).

			**MNI**			
	**Region**	***X***	***Y***	***Z***	**Size**	***t***
R	Middle temporal gyrus	60	−30	−12	197	7.41212416
		50	−26	−12		7.2957921
	Inferior temporal gyrus	52	−16	−30		6.50495815
L	Middle temporal gyrus	−56	−20	−20	23	7.19963741
R	Middle temporal gyrus	50	−42	6	17	6.6976552
R	Angular gyrus	36	−72	38	13	6.42334318

*All results reported as *t*-tests with family-wise error (FWE) correction at *p* < 0.05, cluster size of 10 voxels. Coordinates listed without cluster size belong to the previous listed cluster. Size = number of voxels activated in the cluster.*

## Discussion

Listening to musical rhythms predictably activated the bilateral STG as well as bilateral angular gyrus. Areas in the STG attenuated after the first 4 s of repetition of the rhythmic patterns overlapped exclusively with these areas, with the size of deactivation being larger in the left STG. In addition, activity in bilateral cerebellum, and the (predominantly right) basal ganglia, including caudate nucleus, putamen, and thalamus, decreased after the first 4 s. Since processing of music (Zatorre and Zarate, 2012) and particularly rhythm (Thaut et al., 2014) has been found to be right lateralized (Large et al., 2015), this larger deactivation in the left STG might reflect an asymmetric allocation of resources, where—after initial processing in sensory cortices—the processing of musical rhythms is predominantly done in the right STG. The rapid deactivation of the basal ganglia and cerebellar areas points to a role for these areas in initial beat detection (Peretz and Zatorre, 2005; Grahn, 2009).

During the prolonged listening to rhythms after the initial 16 s of encoding, larger activity was found in the SMA and the caudate nucleus, areas well known to be activated by rhythm (Grahn and Brett, 2007; Bengtsson et al., 2009). Areas related to attention, such as the anterior insula and frontal areas (the inferior frontal gyrus in particular, but also middle and superior frontal areas), were also activated. These areas have been directly implemented in rhythm perception (Chapin et al., 2010; Heard and Lee, 2020), in particular as crucial in working memory for rhythm (Jerde et al., 2011). Activity in the precuneus, the posterior part of the cingulate cortex, and middle prefrontal cortex has in addition been found to play a particular role in the maintenance of musical beats with high beat salience (Toiviainen et al., 2020), which the rhythms in the current study must be characterized as. The areas found to be more activated during maintenance (anterior insula, frontal areas, SMA, posterior cingulate, and precuneus), with more activity in the right hemisphere, were clearly distinct compared to the other conditions and could indicate that working memory mechanisms of representational maintenance are allocated in different areas of the brain, separate from primary cortices activated during initial sensory processing.

Omission activation was distinctly more posterior than listening, suppression, and maintenance, located predominantly in the MTG, and, as expected, omission activation was significantly bigger in the right MTG (Raij et al., 1997) (see Figure 3 and Table 4), with coordinates closely matching those found in previous fMRI (Mustovic et al., 2003; Voisin et al., 2006) and EEG (SanMiguel et al., 2013a, b) studies on omissions and silences in healthy controls and in our previous fMRI study on persons with Parkinson’s disease (Vikene et al., 2019).

In addition, at uncorrected levels, the more salient position of the omission showed a higher activation in the ACC, consistent with previous research implicating the ACC in saliency detection (Menon, 2011) (Supplementary Figure 1 and Supplementary Table 1).

On a theoretical level, our findings can be interpreted as manifestations of crucial principles in predictive processing frameworks, where repetition suppression (Friston, 2005; Baldeweg, 2006; Auksztulewicz and Friston, 2016) can be interpreted as model building; maintenance (Brechmann et al., 2007; Kumar et al., 2016) as a “representation unit” (Clark, 2013); and omission activation (Raij et al., 1997; Wacongne et al., 2011)—an internal response based solely on expectancy or prediction (Jongsma et al., 2005)—as a prediction error (Friston, 2005). Despite the limitations in this study, we will claim that it robustly shows results in line with previous findings on repetition suppression and omission activation—and the perception of musical rhythms in general—and that our paradigm, concretely or abstractly, can be used as a starting point for more refined studies of predictive processing mechanisms for the perception of musical rhythms.

### Limitations

Due to the small sample of participants (*n* = 10) taken from a homogenous population of Western Educated Industrialized Rich Democratic (WEIRD) students of psychology, our findings are difficult to generalize. During a continuous scanning paradigm, the scanner is never silent, which makes the study of omissions questionable, although our results are consistent with previous research. Previous studies have also shown differences in omission detection between musicians and non-musicians (Ono et al., 2013, 2015), but we did not find a difference between them in this study. This might be a result of a low number of participants in the study. Using musical stimuli at “ecological” tempo (120 bpm), with 250 ms ISI in the isochronous metric framework, meant that omissions had to be distributed at fairly long intervals (between 8 and 17.5 s), and to amass sufficient instances of the omissions for statistical analysis, the total scan time for the paradigm was long (33 min). The lengthy paradigm could have affected levels of vigilance and attention during the scan and, as a consequence, could have influenced the results. We did try to remedy this by adding an overt target-detection task, which all participants performed correctly. Furthermore, we only used two rhythms, both of which were repeated several times during the scans. The consequence of longer-term habituation and learning effects could therefore also have influenced the results. In addition, the lack of effects on complexity in our study could indicate that the two rhythms chosen for the study did not differ (enough) in their level of complexity to yield such differences. Finally, due to the poor temporal resolution of fMRI, we might not have been able to pick up finer details of the mechanisms we have tried to describe. Future studies should try to limit paradigm length and use more varied and perhaps “real” musical samples, also with more levels of complexity. Factors such as musical aptitude, level of vigilance, and general working memory capacity should also be taken into consideration in future studies.

## Conclusion

Our study successfully replicated previous findings for repetition suppression and omission activation and shows that tailored musical stimuli can be used in an fMRI setting to robustly investigate such neural phenomena, even with a limited number of participants (*n* = 10). Importantly, our findings show a clear separation between repetition suppression and prediction error activation, and additionally indicate that representational maintenance activates areas different from those deactivated during repetition suppression. While listening and subsequent repetition suppression were located mainly in anterior parts of the superior temporal gyrus, prediction errors (omission activation) were clearly separated from these areas and located mainly in posterior parts of the MTG. Representational maintenance activated SMA, caudate nucleus, anterior insula, frontal areas, and middle and posterior cingulate cortex, potentially showing persistent representational maintenance activity in areas separate from initial listening (encoding), repetition suppression (attenuation), and prediction error (omission).

## Data Availability Statement

The raw data supporting the conclusions of this article will be made available by the authors, without undue reservation.

## Ethics Statement

The studies involving human participants were reviewed and approved by Regional Committee for Medical and Health Research Ethics, REK-VEST (2014/1915). The patients/participants provided their written informed consent to participate in this study.

## Author Contributions

UF, KS, and KV contributed in the conception of the research project, did statistical analysis, and contributed to the final version of the manuscript. UF recruited the participants, organized the study, and wrote the first draft. KV designed the paradigm. All authors contributed to the article and approved the submitted version.

## Conflict of Interest

The authors declare that the research was conducted in the absence of any commercial or financial relationships that could be construed as a potential conflict of interest.

## Publisher’s Note

All claims expressed in this article are solely those of the authors and do not necessarily represent those of their affiliated organizations, or those of the publisher, the editors and the reviewers. Any product that may be evaluated in this article, or claim that may be made by its manufacturer, is not guaranteed or endorsed by the publisher.

## Supplementary Material

Supplementary Figure 1 Visualization of findings in Supplementary Table 1.

Supplementary Table 1 Omission Position 1 > Position 2 across rhythms. Results reported as *t*-tests uncorrected at *p* < 0.001, cluster size of 100 voxels. ACC, anterior cingulate cortex; MPFC, medial prefrontal cortex.
