# Supplementary figures and images for: Suppression, Maintenance, and Surprise: Neuronal Correlates of Predictive Processing Specialization for Musical Rhythm
Source: Front Neurosci. 2021 Aug 27;15:674050. doi: 10.3389/fnins.2021.674050 (PMC8429816; doi:10.3389/fnins.2021.674050)

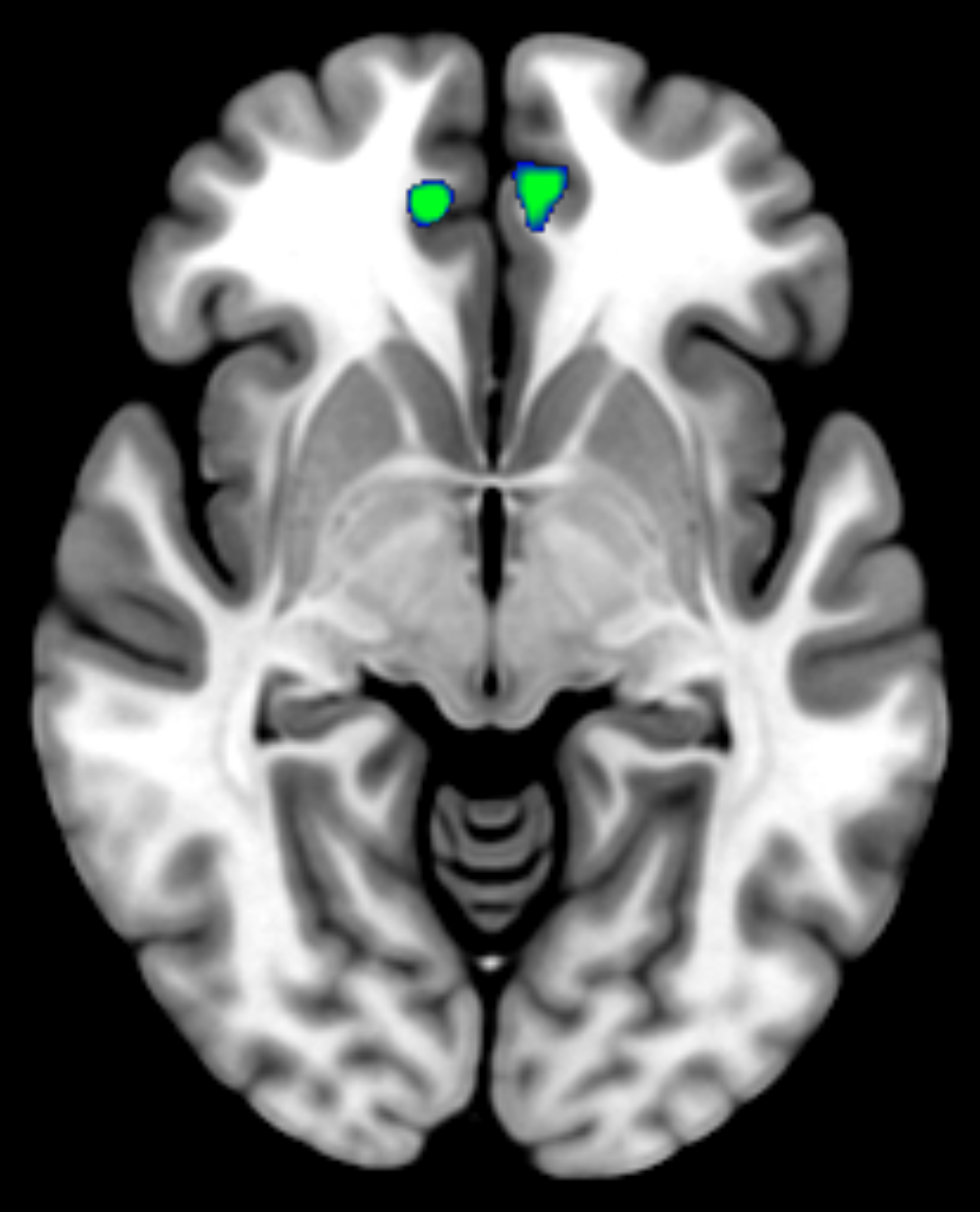

Supplement: Supplementary Figure 1 — Visualization of findings in Supplementary Table 1. [file Image_1.TIF]
