# Supplementary material for: Suppression, Maintenance, and Surprise: Neuronal Correlates of Predictive Processing Specialization for Musical Rhythm
Source: Front Neurosci. 2021 Aug 27;15:674050. doi: 10.3389/fnins.2021.674050 (PMC8429816; doi:10.3389/fnins.2021.674050)
Supplement: Supplementary Table 1 — Omission Position 1 > Position 2 across rhythms. Results reported as t-tests uncorrected at p < 0.001, cluster size of 100 voxels. ACC, anterior cingulate cortex; MPFC, medial prefrontal cortex. [file Table_1.DOCX]

| Supplementary Table 1: Omission Position 1 > Position 2 across rhythms | | | | | | |
| --- | --- | --- | --- | --- | --- | --- |
|  |  |  | MNI |  |  |  |
|  | Region | X | Y | Z | Size | t |
| R | Middle Frontal Cortex, Orbital | 8 | 46 | -6 | 367 | 4.34 |
| L | Anterior Cingulum Cortex | -10 | 50 | -2 |  | 4.08 |
| L | Anterior Cingulum Cortex | -4 | 52 | 8 |  | 3.88 |
|  |  |  |  |  |  |  |
| Supplementary Table 1 legend: Results reported as t-tests uncorrected at p<0.001, cluster size of 100 voxels. Size = Number of voxels activated in the cluster. | | | | | | |
